# Radical surgery for stage IB2/IIA2 cervical cancer: A large retrospective study

**DOI:** 10.3389/fonc.2022.948298

**Published:** 2022-09-23

**Authors:** Fang Zhou, Xuedong Tang, Zhuyan Shao, Xi Chen, Wen Gao, Chenyan Fang, Zejia Wang, Ping Zhang, Tao Zhu, Huarong Tang

**Affiliations:** ^1^ Department of Gynecological Oncology, The Cancer Hospital of the University of Chinese Academy of Sciences, Zhejiang Cancer Hospital, Institute of Basic Medicine and Cancer (IBMC), Chinese Academy of Sciences, Hangzhou, China; ^2^ School of the Second Clinical Medical College, Zhejiang Chinese Medical University, Hangzhou, China; ^3^ Department of Gynecology, Jiaxing University Affiliated Women and Children Hospital, Jiaxing, China; ^4^ The Cancer Hospital of the University of Chinese Academy of Sciences, Zhejiang Cancer Hospital, The Key Laboratory of Zhejiang Province for Aptamers and Theranostics, Institute of Basic Medicine and Cancer (IBMC), Chinese Academy of Sciences, Hangzhou, China; ^5^ Kent School, Kent, CT, United States; ^6^ Department of Gynecological Radiotherapy, The Cancer Hospital of the University of Chinese Academy of Sciences, Zhejiang Cancer Hospital, Institute of Basic Medicine and Cancer (IBMC), Chinese Academy of Sciences, Hangzhou, China

**Keywords:** bulky early-stage cervical cancer, stage IB2, stage IIA2, radical surgery, adjuvant therapy

## Abstract

**Background:**

We aimed to evaluate survival, complications, and prognostic factors in patients with IB2/IIA2 (FIGO 2009, bulky early-stage) cervical cancer (CC) who were primarily treated with radical surgery (RS).

**Methods:**

From January 2011 to January 2018, patients with stage IB2/IIA2 CC who underwent RS ± adjuvant therapy were enrolled and retrospectively evaluated. Survival was estimated using the Kaplan–Meier method. Significance was determined using the log-rank test. Multivariate regression analyses were performed to determine prognostic factors.

**Results:**

Of the 975 enrolled patients, 877 (89.9%) received adjuvant therapy. The median follow-up was 48 months, the 5-year overall survival (OS) was 85.9%, and the 5-year progression-free survival (PFS) rate was 80.8%. Multivariate analysis showed that histological type, pelvic lymph nodes, and para-aortic lymph nodes were independent prognostic factors for PFS and OS. Tumor diameter was also an independent prognostic factor with OS. Recurrent disease developed in 14.3% (140) of patients., including local, distant, and both recurrences in 55 (5.6%), 71 (7.3%), and 14 (1.4%) patients, respectively. Grade 3–4 short-term complications occurred in 196 (20.1%) patients, and long-term complications occurred in 86 (8.8%) patients. Short-term hematological complications occurred in 99 cases (10.2%). No significant differences in non-hematological complications were detected between the RS and RS + adjuvant therapy groups.

**Conclusions:**

RS followed by adjuvant therapy is a feasible and effective treatment for IB2/IIA2 CC, with a high 5-year survival rate and an acceptable incidence of complications. Positive pelvic lymph nodes and para-aortic abdominal lymph nodes significantly impact PFS and OS. Evaluation of lymph node status before surgery is important. RS is recommended for patients with negative lymph node metastasis.

## Introduction

Cervical cancer (CC) is a common malignant tumor of the female genital tract, which seriously threatens women’s health. In 2018, 569,847 new cases of CC and 311,365 deaths occurred wordwide (1). In China, 119,300 new cases of CC and 37,200 deaths were reported in 2016 (2). The recurrence and mortality rates of bulky early-stage (FIGO 2008 stage IB2/IIA2) CC are higher than those of early-stage (stage IB1/IIA1) CC (recurrence rates: 34% vs. 20%; 5-year OS rates: 70% vs. 87%) (3–5). According to the National Comprehensive Cancer Network (NCCN) guidelines, treatment for bulky early-stage CC includes primary chemoradiotherapy (CCRT) (category 1: based upon high-level evidence and a uniform NCCN consensus that the intervention is appropriate) and radical surgery (RS) followed by adjuvant therapy (category 2B: based upon lower-level evidence and a NCCN consensus that the intervention is appropriate) (6). The evidence for CCRT is mainly based on five prospective studies published in the 1990s. In addition, Landoni (7) reported that the survival outcomes of RS and radiotherapy (RT) were similar in IB2/IIA2 CC patients in 1997. In recent years, three retrospective studies have shown that survival after RS followed by adjuvant therapy was better than survival after CCRT (8–10). In 2020, Liu showed that 5-year OS rates for RS were better than those for CCRT in a long-term oncological outcome analysis of IB1 to IIA2 cervical cancer patients from 37 Chinese hospitals (81.5% vs. 72.5%, P = 0.039) (8).

Large study data are still lacking to support RS as an alternative treatment modality. Therefore, the survival, complications, and prognostic factors in patients with bulky early-stage CC who underwent RS ± adjuvant therapy were retrospectively analyzed at our hospital. The current study is the largest data so far. The main outcome in this analysis was overall survival (OS), and the secondary outcomes were progression-free survival (PFS) and complications.

## Materials and methods

### Patients

**Figure 1:**
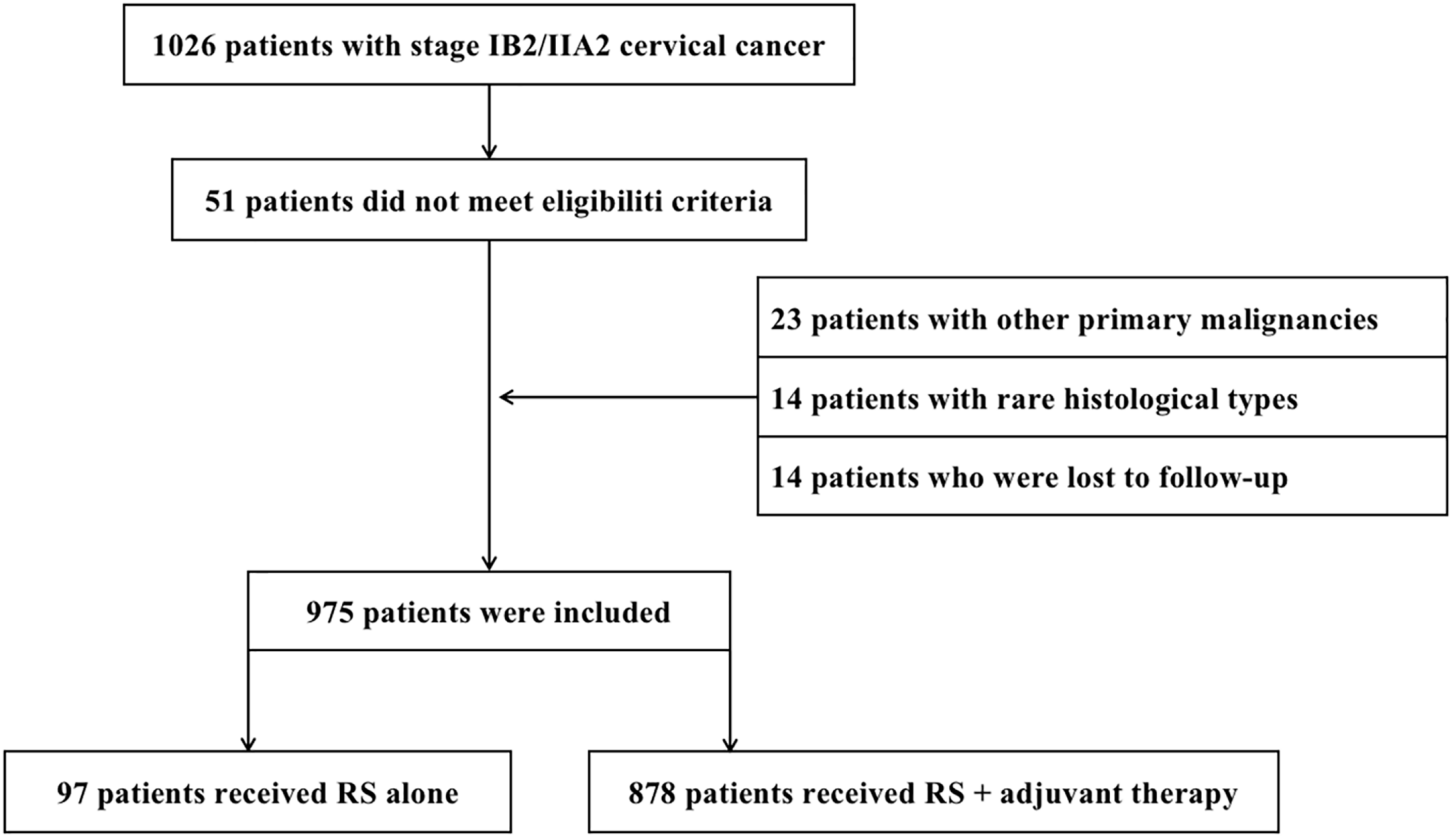
Data screening process.

This retrospective study was approved by the Ethics Committee of the Cancer Hospital of the University of Chinese Academy of Sciences (approval number IRB-2021-116, approved on 16 April 2021). Informed consent was waived for this retrospective study. From January 2011 to January 2018, 1,026 patients with bulky early-stage CC underwent RS at the Cancer Hospital of the University of Chinese Academy of Sciences. Twenty-three patients with other primary malignancies, 14 patients with rare histological types, and 14 patients who were lost to follow-up were excluded from the analysis. Hence, 975 patients were included in the study (**Figure 1**). Inclusion criteria were as follows: (1) squamous cell carcinoma (SCC), adenocarcinoma (AC), and adenosquamous carcinoma (ASC) confirmed by pathological results after surgery; (2) complete clinical data; (3) stage IB2 or IIA2 according to the International Federation of Gynecology and Obstetrics (FIGO 2009); and (4) underwent RS ± adjuvant therapy. Patients were excluded based on the following criteria: (1) underwent CCRT/RT as initial treatment; (2) rare histological types (including small cell neuroendocrine carcinoma, clear cell carcinoma, carcinosarcoma, and endometrioid adenocarcinoma); (3) other primary malignancies; and (4) lost to follow-up (actual follow-up time was no more than 6 months). The following data were retrieved from the hospital medical record system: age, FIGO stage, neoadjuvant chemotherapy, operation method (laparotomy or laparoscopy), operation duration (min), blood loss (mL), blood transfusion (mL), para-aortic abdominal lymph node resection, ovarian transposition, adjuvant therapy, histological type, differentiation grade, depth of stromal invasion, lymph-vascular space invasion (LVSI), cervical tumor diameter, lymph node metastasis (LNM), the state of resection margin, parametrial involvement, complications, site of recurrence, and follow-up status.

### Treatment

RS included Querleu–Morrow type C2 radical hysterectomy and bilateral pelvic lymph node dissection including common, internal, and external iliac lymph nodes, deep inguinal lymph nodes, and obturator lymph nodes. Radical hysterectomy was systematically performed, followed by pelvic lymph node dissection. Bilateral common iliac lymph nodes underwent intraoperative frozen section examination for rapid pathological examination. Indications for para-aortic abdominal lymph node dissection included enlarged para-aortic lymph nodes found during intraoperative exploration and common iliac lymph nodes confirmed to be positive by rapid pathological examination of intraoperative frozen sections. Biopsy was indicated if the cervical tumors were larger than 4 cm. The NCCN guidelines recommend para-aortic abdominal lymph node dissection at the level of the inferior mesenteric artery (higher extent of resection if necessary), with the abdominal aortic bifurcation as the lower boundary (6). The extent of clearance included lymph nodes and adipose tissue between the inferior vena cava and the abdominal aorta and between the anterior and posterior left and right sides.

After surgery, patients with any high-risk factors including LNM, parametrial involvement, and positive resection margins received adjuvant CCRT. Those with two or more intermediate-risk factors including LVSI, deep stromal invasion, and tumor diameter >4 cm received adjuvant CCRT/RT before 2015. According to the Sedlis criteria, patients received adjuvant CCRT/RT after 2015 (11) (**Table 1**). Adjuvant RT was initiated 4~6 weeks after RS. Three-dimensional conformal RT or intensity-modulated RT in the pelvic field, with a radiation dose of 4,500~5,040 cGy/25~28 fractions, was administered once a day, five times a week. If the common iliac or para-aortic abdominal lymph nodes were positive, RT of the abdominal and pelvic joint fields was also performed. Patients with positive vaginal margins received brachytherapy. Patients who were eligible for adjuvant chemotherapy received 40 mg/m^2^ of cisplatin weekly during the course of adjuvant RT for 4~5 cycles.

**Table 1:** Sedlis criteria for external pelvic radiation after radical hysterectomy in node-negative, margin-negative, parametria-negative cases.

LVSI	Stromal invasion	Tumor size (cm)
+	Deep 1/3	Any
+	Middle 1/3	≥2
+	Superficial	≥5
–	Middle or deep 1/3	≥4

LVSI, lymph-vascular space invasion.

### Follow-up

Patients were followed up every 3 months for the first 2 years, every 6 months up to 5 years, and annually thereafter. Followed-up examinations included gynecological examinations, transvaginal ultrasonography, vaginal vault cytology, and measurement of serum tumor markers. When recurrent disease was suspected, diagnostic tests such as MRI, CT scans, or PET-CT, and/or biopsies were performed. OS was defined as the period from surgery to death or last follow-up. PFS was defined as the period from surgery to recurrence or last follow-up. The site of recurrence was categorized as local (relapse in the vaginal stump or abdominal region) or distant (relapse in areas outsides the abdomen, including organs and lymph nodes). Based on time after treatment, complications were categorized into short-term (within 4 weeks) and long-term (after 4 weeks) complication. Complications were determined based on the National Cancer Institute Common Terminology Criteria for Adverse Events version 4.0 (12) and graded according to the Clavien–Dindo classification criteria (13). Complications were divided into five grades; grade 0~2 complications were mild and did not require special intervention. Mild complications were not analyzed in this study. Hematological complications included anemia, leukopenia, neutrophil count decrease, and platelet count decrease. Non-hematological complications included all other complications not related to the hematologic system.

### Statistical analyses

SPSS 26.0 software (IBM Corp., Armonk, NY, USA) was used for statistical analyses. Data were tested for normality, and data conforming to normal distribution were expressed as means ± standard errors (x ± s). Data that were not normally distributed were expressed as medians with ranges (M, P25–P75). Fisher’s exact or chi-square tests were used to evaluate the categorical results. Survival rates were calculated, and survival curves were plotted using the Kaplan–Meier method. Relevant clinicopathological factors affecting patients prognosis were analyzed using the log−rank test for univariate analysis and the Cox regression for multivariate analysis. P < 0.05 indicated statistical significance.

## Results

### Clinicopathological characteristics

The clinicopathological characteristics of the patients are shown in **Table 2**. The median patient age was 50 years. During surgeries, 28.5% (278) of the patients underwent para-aortic abdominal lymph node dissection and 24.7% (241) patients underwent ovarian transposition. The median operating duration was 170 min (60–420 min), the median blood loss was 200 ml (0–2020 ml), and 22.6% of patients required blood transfusions. No perioperative mortality occurred.

**Table 2:** Clinicopathological characteristics.

Characteristics	N	%
Stage		
IB2	422	43.3
IIA2	553	56.7
Surgical approach		
Laparotomy	953	97.7
Laparoscopy	22	2.3
Neoadjuvant chemotherapy		
Yes	27	2.8
No	948	97.2
Lymph node dissection		
Pelvic	697	71.5
Pelvic + para-aortic	278	28.5
Ovarian transposition		
No	724	75.3
Yes	241	24.7
Blood transfusion	220	22.6
Adjuvant therapy		
None	98	10.1
Chemoradiotherapy	680	67.7
Radiotherapy	180	18.5
Chemotherapy	17	1.7
Histological type		
SCC	864	88.6
AC/ASC	111	11.4
Histological grade		
Low	484	49.6
Intermediate	486	49.8
High	5	0.5
Pelvic lymph nodes		
Positive	393	40.3
Negative	582	59.7
Para-aortic lymph nodes		
Positive	58	5.9
Negative	917	94.1
Parametrial involvement		
Yes	37	3.8
No	938	96.2
Surgical margins		
Positive	9	0.9
Negative	966	99.1
Tumor diameter, cm		
4–5.9	701	71.9
≥6	274	28.1
LVSI		
Yes	554	56.8
No	421	43.2
Depth of stromal invasion		
<1/3	75	7.7
1/3–2/3	196	20.1
>2/3	704	72.2

AC, adenocarcinoma; ASC, adenosquamous carcinoma; LVSI, lymph-vascular spaceinvasion; SCC, squamous cell carcinoma.

SCC was detected in 88.6% of patients, and 11.4% had AC/ASC. Positive pelvic lymph nodes were present in 40.3% (393) patients. Positive para-aortic abdominal lymph nodes were detected in 5.9% (58) of patients including 51 patients who underwent resections and seven patients who underwent biopsies. Thirty-seven patients (3.8%) had parametrial involvement, and 0.9% (9) patients had positive resection margins. The median cervical tumor diameter was 5.0 cm (4.1~11 cm). Deep stromal invasion occurred in 72.2% (704) patients, and 56.8% (554) of patients had LVSI. Ninety-eight patients (10.1%) received no adjuvant therapy, 67.7% (680) received CCRT, 18.5% (180) received RT alone, and 1.7% (17) received chemotherapy alone.

### Survival and prognostic factors

**Figure 2:**
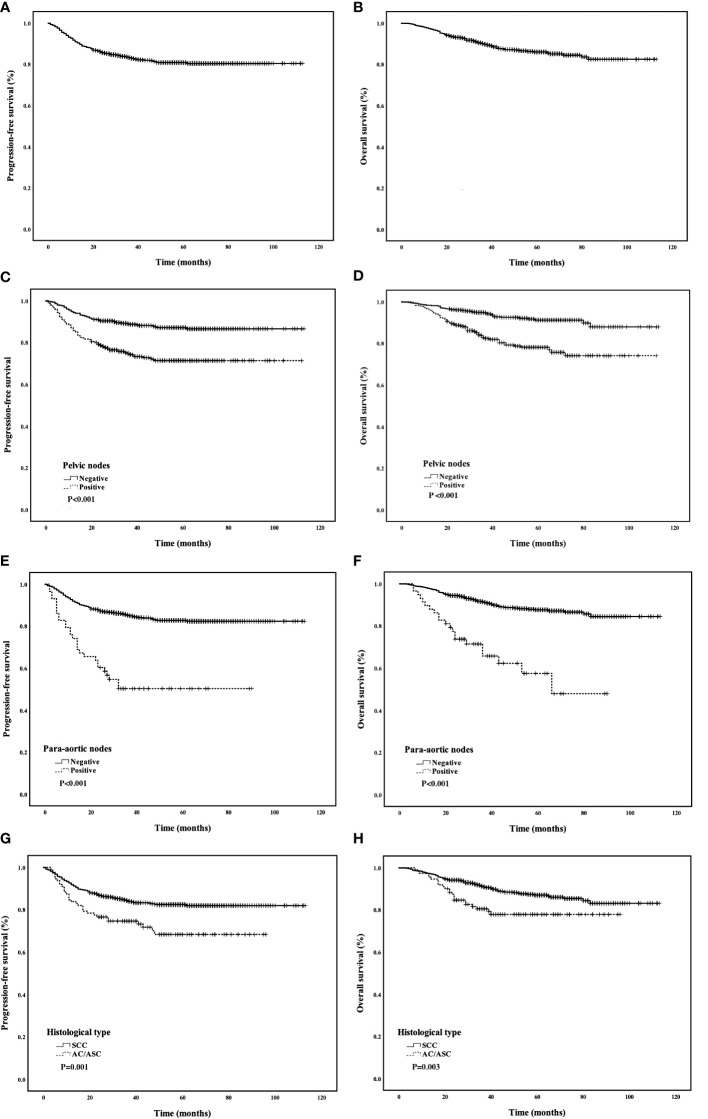
**(A, B)** PFS and OS of all patients; **(C, D)** Comparison of PFS and OS curves between different histological types; **(E, F)** Comparison of PFS and OS curves between positive and negative pelvic lymph nodes; **(G, H)** Comparison of PFS and OS curves between positive and negative para-aortic lymph nodes.

The median follow-up was 48 months, and 117 patients (12.0%) died. The 5-year PFS was 80.8%, and the 5-year OS was 85.9% (**Figures 2A** **, B**). Univariate analyses showed that FIGO stage, tumor diameter, histological type, parametrial involvement, pelvic lymph nodes, para-aortic lymph nodes, deep stromal invasion, and LVSI were associated with PFS and OS (P < 0.05) (**Table 3**). Multivariate analysis showed that histological type (HR = 1.90, 95% CI = 1.20–3.01, P = 0.007), pelvic lymph nodes (HR = 1.89, 95% CI = 1.21–2.93, P = 0.005), and para-aortic lymph nodes (HR = 2.28, 95% CI = 1.36–3.85, P = 0.002) were independent prognostic factors for PFS. Histological type (HR = 1.78, 95% CI = 1.21–2.63, P = 0.004), pelvic lymph nodes (HR = 1.61, 95% CI = 1.13–2.29, P = 0.009), para-aortic lymph nodes (HR = 2.20, 95% CI = 2.20–1.41, P = 0.001), and tumor diameter (HR = 1.68, 95 CI = 1.15–2.46, P = 0.007) were independent prognostic factors for OS (**Table 4**). The 5-year PFS and OS rates for SCC (85.5% and 87.0%) were significantly higher than the rates for AC/ASC (73.8% and 77.8%) (P = 0.009 and 0.016, respectively) (**Figures 2C** **, D**). The 5-year OS rates for patients with tumor diameters of 4.1–6 cm and ≥6 cm were 88.8% and 79.7%, respectively (P = 0.002). The 5-year PFS and OS rates for positive pelvic lymph nodes (71.3% and 78.1%) were significantly higher than the rates for negative pelvic lymph nodes (87.1% and 91.2%) (P < 0.001 and P < 0.001,respectively) (**Figures 2E, F**). The 5-year PFS rates in patients with positive and negative para-aortic lymph nodes were 50.3% and 82.7% (P<0.001), and the 5-year OS rates were 57.5% and 87.6%, respectively (P<0.001) (**Figures 2G, H**).

**Table 3:** Univariate analysis of PFS and OS.

	PFS		OS	
	5-year PFS (%)	P-value	5-year OS (%)	P-value
Age, years		0.239		0.289
<40	86.8		89.9	
40–59	80.2		86.1	
≥60	76.8		81.5	
Stage		0.049		0.011
IB2	83.4		89.5	
IIA2	78.8		82.9	
Tumor diameter, cm		0.037		<0.001
4–5.9	82.3		88.8	
≥6	77.1		79.7	
Histological grade		0.904		0.506
Low	80.7		87.3	
Intermediate	81.0		84.8	
High	80.0		100	
Histological type		0.001		0.003
SCC	82.4		87.0	
AC/ASC	68.3		77.8	
Parametrial involvement		0.003		0.002
No	81.5		86.6	
Yes	61.4		69.7	
Positive resection margin		0.226		0.297
No	80.9		86.0	
Yes	66.7		76.2	
Pelvic lymph nodes		<0.001		<0.001
Negative	87.1		91.2	
Positive	71.3		78.1	
Para-aortic lymph nodes		<0.001		<0.001
Negative	82.7		87.6	
Positive	50.3		57.9	
Depth of stromal invasion		<0.001		0.001
<1/3	96.0		98.7	
1/3–2/3	86.6		90.4	
>2/3	71.5		82.9	
LVSI		<0.001		0.003
No	87.2		90.2	
Yes	76		82.8	

AC, adenocarcinoma; ASC, adenosquamous carcinoma; LVSI, lymph-vascular space invasion; PFS, progression-free survival; OS, overall survival; SCC, squamous cell carcinoma.

**Table 4:** Multivariate analysis of PFS and OS.

		PFS			OS	
	HR	95% CI	P-value	HR	95% CI	P-value
Stage (IB2 vs. IIA2)	1.37	0.93–2.02	0.109	1.21	0.89–1.66	0.223
Tumor diameter, cm (4–5.9 vs. ≥6)	1.87	0.94–1.79	0.116	1.68	1.15-2.46	0.007
Histological type (AC/ASC vs. SCC)	1.90	1.20–3.01	0.007	1.78	1.21–2.63	0.004
Parametrial (no vs. yes)	1.21	0.63–2.35	0.563	1.17	0.64–2.11	0.612
Pelvic lymph nodes (negative vs. positive)	1.89	1.21–2.93	0.005	1.61	1.13–2.29	0.009
Para-aortic lymph nodes (negative vs. positive)	2.28	1.36–3.85	0.002	2.20	2.20–1.41	0.001
Depth of stromal invasion			0.072			0.06
<1/3 vs. 1/3–2/3	5.00	0.66–37.65	0.118	2.81	0.85–9.30	0.091
<1/3 vs. >2/3	7.15	0.98–52.04	0.052	3.60	1.13–11.47	0.030
LVSI (no vs. yes)	1.25	0.80–1.93	0.325	1.39	0.97–1.99	0.072

AC, adenocarcinoma; ASC, adenosquamous carcinoma; CI, confidence interval; HR, hazard ratio; LVSI, lymph vascular space invasion; PFS, progression-free survival; OS, overall survival; HR, hazard ratio; CI, confidence interval; SCC, squamous cell carcinoma.

### Patterns of failure

Recurrent disease developed in 140 (14.3%) patients including local recurrence in 55 (5.6%) patients, distant recurrence in 71 (7.3%) patients, and both in 14 (1.4%) patients. The overall recurrent rates were similar in patients who underwent RS alone and patients who underwent RS + adjuvant treatment (16.7% vs. 14.1%, P = 0.527). However, the local recurrence rate was significantly higher in the RS-alone group compared with the rate in the RS + adjuvant treatment group (11.3% vs. 5.0%, P = 0.01). No significant differences in distant metastases and local recurrence + distant metastases were detected between the two groups (3.1% vs. 7.7%, P = 0.094; 2.1% vs. 1.4%, P = 0.585) (**Table 5**).

**Table 5:** Patterns of failure.

	Total	No. of patients (%)		
		RS alone (n = 96)	RS + adjuvant therapy (n = 878)	P-value
Recurrence	140 (14.3)	16 (16.7)	124 (14.1)	0.527
Local	55 (5.6)	11 (11.3)	44 (5.0)	0.01
Distant	71 (7.3)	3 (3.1)	68 (7.7)	0.094
Both	14 (1.4)	2 (2.1)	12 (1.4)	0.585

### Complications

Among the 975 patients with CC, 263 (27%) patients had grade 3–4 complications after treatment, including 196 (20.1%) patients with short-term complications and 86 (8.8%) patients with long-term complications. Among the patients with short-term complications, 99 (10.2%) patients had hematological complications and 110 (11.3%) patients had non-hematological complications. The incidence of grade 3~4 complications was significantly higher in the RS + adjuvant treatment group compared with the incidence in the RS-alone treatment group (28.4% vs. 14.4%, P = 0.011). The incidence of grade 3~4 short-term non-hematological complications + long-term complications was 19.7% in patients undergoing RS + adjuvant therapy and 14.4% in patients undergoing RS alone (P = 0.211) (**Table 6**). All 99 patients who experienced grade 3~4 short-term hematological complications belonged to the RS + adjuvant treatment group, and patients returned to normal after symptomatic and supportive treatment.

**Table 6:** Complications in different treatment groups.

	T	Surgery alone (n = 97) No (%)	Surgery + adjuvant therapy (n = 878) No (%)	P-value
Short-term complications	196	9 (9.3)	187 (21.3)	0.025
Hematology	99	–	99 (11.3)	–
Non-hematology	110	9 (9.3)	101 (11.5)	0.511
Long-term complications	86	5 (5.2)	81 (9.2)	0.18
Total	263	14 (14.4)	249 (28.4)	0.011
Other total^a^	187	14 (14.4)	173 (19.7)	0.211

Patients with both short-term and long-term complications were counted as early and late complications, respectively; hematological and other complications were counted as corresponding types of complications. ^a^means short-term non-hematological complications + long-term complications; - means none.

Intraoperative complications occurred in 112 (11.5%) patients; complications included bladder injury in 43 (4.4%) patients, ureteral injury in 28 (2.9%) patients, bowel injury in six (0.6%) patients, and vascular injury in 35 (3.6%) patients. These complications were all grade 2. In the RS + adjuvant treatment group, 187 (21.3%) patients had short-term complications, including 99 myelosuppression, 32 intestinal obstruction, and 22 lymphocele cases, and 81 (9.2%) patients had long-term complications, including 37 lymphocele, 23 urinary tract obstruction, and eight urinary fistula cases. In the RS alone, nine (9.3%) patients had short-term complications, including two intestinal obstruction, two lymphocele cases, and one each of pulmonary infection, urinary fistula, and incision dehiscence cases. Five (5.2%) patients in the RS-alone treatment group experienced long-term complications, including urinary tract obstruction in three patients and lymphocele and bladder dysfunction in one patient each (**Table 7**).

**Table 7:** Grade 3–4 complications after surgery alone and surgery + adjuvant therapy in patients with cervical cancer.

	Short-term complications	Number of patients (%)	Long-term complications	Number of patients (%)
RS alone		9 (9.3)		5 (5.2)
	Intestinal obstruction	2 (2.1)	Urinary tract obstruction	3 (3.1)
	Lymphocele t	2 (2.1)	Bladder dysfunction	1 (1.0)
	Urinary tract infection	2 (2.1)	Lymphocele	1 (1.0)
	Urinary fistula	1 (1.0)	–	–
	Incision dehiscence	1 (1.0)	–	–
	Lung infection	1 (1.0)	–	–
RS + adjuvant		187 (21.3)		81 (9.2)
	Myelosuppression	94 (10.7)	Lymphocele	37 (4.2)
	Intestinal obstruction	32 (3.6)	Urinary tract obstruction	23 (2.6)
	Lymphocele	22 (2.5)	Urinary fistula	8 (0.9)
	Urinary tract infection	18 (2.1)	Bladder voiding dysfunction	4 (0.5)
	Myelosuppression + urinary tract infection	5 (0.6)	Incisional hernia	3 (0.3)
	Urinary fistula	4 (0.5)	Abdominal infection	2 (0.2)
	Incision dehiscence	4 (0.5)	Intestinal obstruction	1 (0.1)
	Abdominal infection	2 (0.2)	Lymphocyst + urethral obstruction	1 (0.1)
	Pulmonary embolism	2 (0.2)	Lymphocyst + urinary tract infection	1 (0.1)
	Lower extremity venous thrombosis	1 (0.1)	Lymphocyst + lower-extremity venous thrombosis	1 (0.1)
	Intestinal fistula	1 (0.1)	–	–
	Intestinal obstruction + urinary tract infection	1 (0.1)	–	–
	Intestinal obstruction + abdominal infection	1 (0.1)	–	–

- means none.

### Stratified analysis according to lymph node status

Stratified analysis based on the lymph node status showed that the 5-year PFS rates were 87.4%, 76%, and 50.3% (P < 0.001) and the 5-year OS rates were 91.7%, 82.4%, and 57.5% (P < 0.001) in the lymph node-negative, pelvic lymph node-positive, and para-aortic lymph node-positive groups, respectively. Adjuvant therapy was significantly higher in patients with positive pelvic and para-aortic lymph nodes compared to patients with negative lymph nodes (92.6% vs. 88.3%, P = 0.026). In addition, patients with positive pelvic or para-aortic lymph nodes had more overall complications (31.5% vs. 23.9%, P = 0.009) and more hematological complications (14.2% vs. 7.4%, P = 0.001) compared to patients with negative lymph nodes. In the para-aortic lymph node-positive, pelvic lymph node-positive, and lymph node-negative groups, 29.3%, 19.3%, and 18.1% experienced non-hematological complications, respectively; non-hematological complications occurred significantly more frequently in the para-aortic lymph node-positive group compared with the lymph node-negative group (P = 0.038).

## Discussion

We reviewed the clinical data of 975 patients with bulky early-stage CC who underwent RS ± adjuvant therapy and analyzed the survival, failure patterns, and complications in this large Chinese retrospective study. In the 1990s, the Gynecologic Oncology Group (GOG) launched five randomized controlled trials on CCRT for locally advanced CC (14–18). Two of the trials reported survival outcomes for patients with stage IB2/IIA2 CC (14, 15). In the study conducted by Morris et al. (15), the 5-year OS rates of 130 patients with stage IB2/IIA2 receiving CCRT or RT were 77% and 58%, respectively. In a study by Keys et al. (14), 368 patients with stage IB2 CC receiving CCRT or RT alone had 3-year OS rates of 83% and 74%, respectively. A randomized controlled trial from Italy in 1997 showed that the 5-year OS rate for stage IB2/IIA2 CCs was similar in patients treated with RS and patients treated with RT (70% vs. 72%) (7). However, these studies were all conducted in the twentieth century. Several recent studies reported that survival outcomes after radical RT/CCRT were lower than outcomes after RS. In 2020, a multicenter retrospective study in Chinese patients with IB2 and IIA2 CC found that the 5-year OS rates were significantly better in patients treated with RS compared with the rates in patients treated with RT (81.5% vs. 72.5%, P = 0.039) (8). Rungrugang et al. (9) also demonstrated that RS treatment in patients with bulky early-stage CC was better than RT treatment (median OS: 75 vs. 67 m, P = 0.001) and disease-specific survival improved (72 vs. 61.4 m, P < 0.0001). Our study agrees with these recent studies; the 5-year OS rate of patients with stage IB2/IIA2 CC who received RS ± adjuvant therapy (85.9%) was significantly higher than the OS rate in patients treated with CCRT in the above study. In addition, Zhu Anna et al. (19) showed that the 2-year disease-free survival rate of patients with stage IB2/IIA2 CC who underwent RS was as high as 89.2%, further supporting our results.

The survival rates of patients in this study were better than the survival rates of previous studies. There are several possible reasons for the improved survival. (1) Doctors are aware of the indications for surgery, especially for parametrial invasion. In this study, the rate of postoperative parametrial involvement was only 3.8% (37/975), highlighting our strictness in case selection. (2) The gynecology department of our hospital has accumulated decades of experience in CC surgery, with an annual number of radical hysterectomied for C2 type of 500–800. For patients with tumor diameters >4 cm, our surgical team advocates fully freeing the space and dissecting the relevant blood vessels and ligaments, while a “rectangular”-type resection of parametrial and paravaginal tissues ensures relatively safe margins. This is also indicated by the postoperative pathological reports showing a positive margin as low as 0.9% (9/975). Furthermore, the local recurrence rate is as low as 5.6% (55/975). (3) Adjuvant radiotherapy and chemotherapy with sufficient doses reduce the recurrence rate.

RS is aimed at removing the primary cervical lesion and surrounding tissue that may be involved, thereby reducing the risk of local recurrence and distant metastasis (20). In this study, 14.3% of patients relapsed, similar to the results of previous studies involving bulky early-stage CC treated with RS (10%–24.7%) (21–23). Keys et al. (14) reported recurrence rates of 21% and 37% in patients with stage IB2 CC received with CCRT and RT alone, respectively, which were higher than the recurrence rates in this study. Landoni et al. (7) also reported a lower recurrence rate for patients with bulky early-stage CC treated with RS compared with the rate in patients treated with RT (34% vs. 42%). For bulky early-stage CC, the recurrence rates after RS are lower than the recurrence rates after RT/CCRT and may be related to the limitations of RT. Local control of RT decreases with increased tumor diameter (24). In addition, we found that the pelvic recurrence rate in patients treated with RS + adjuvant was significantly lower compared with the recurrence rate in patients treated with RS alone (5.0% vs. 11.3%, P = 0.01), indicating that RS combined with adjuvant therapy is better for bulky early-stage CC. RS has several advantages compared with RT. (1) More accurate staging and pathological characteristics can be obtained during RS, which can guide the selection of postoperative adjuvant treatment and evaluate the prognosis (20). (2) For young patients, RS can reduce the damage to ovarian function (25). (3) Complications such as vaginal fibrosis and vaginal fistula caused by vaginal brachytherapy can be avoided; thus, the patient quality of life is better after RS than the quality of life after RT (26).

In 1997, a prospective controlled study showed that the incidence of grade 2–3 complications in patients with bulky early-stage CC treated with RS + adjuvant radiotherapy was higher than the incidence of complications in patients treated with RT (24% vs. 11%) (7). This study suggested that adjuvant treatment after RS for bulky early-stage CC may cause more complications. However, our data demonstrate that the non-hematological complications in patients treated with RS + adjuvant therapy were not statistically different from the complications after RS treatment (19.5% vs. 13.4%, P = 0.147), similar to the results of Laura et al. (27) and Landoni et al. (7). The study by Laura et al. showed that the incidence of major complications in patients with bulky early-stage CC who received RS + adjuvant therapy was not higher than the incidence of complications after surgery alone (4.6% vs. 18%, P = 0.161). The Landoni et al. study found that the incidence of complications in the RS + adjuvant therapy group was not higher than the incidence in the RS group (24% vs. 33%). The GOG 92 study showed that the incidence of grade 3–4 non-hematological complications in the adjuvant treatment group was slightly higher than the incidence in the surgery-only group (7.8% vs. 2.1%) (18), but both groups had a lower incidence of grade 3–4 non-hematological complications than the incidence in the radical RT group from the study above(11%) (7). On the other hand, we found that short-term complications after RS were more common than long-term complications (19.9% vs. 8.8). Among the short-term complications, early hematological toxicity occurred in 10.2% of patients, and patients recovered after symptomatic treatment. Eifel et al. (28) found that the incidence of major complications in the 5 years after radical RT was 9%, and the rate increased to 14% in the 20 years after radical RT. A review of CC involving 1,243 patients from 42 centers showed that the incidence grade 3–4 long-term complication after CCRT was 10%, while the incidence after RS + adjuvant RT was 5% (29). These data indicate that complications after radical CCRT/RT appear later but last longer, resulting in a greater impact on the quality of life.

In this study, histological type and LNM were independent risk factors for survival and recurrence in patients with bulky early-stage CC after RS, which is consistent with the results of Landoniet et al. (7) and Noriaki et al. (23). We also found that tumor diameter is an independent risk factor for survival. The 5-year OS rates were 79.7% and 88.8% in patients with ≥6- and 4–5.9-cm tumors, respectively (HR = 1.68, P = 0.002). A population study based on the SEER database found that RS improved survival by 49% compared with radical RT in patients with 4–6-cm tumors, while in women with tumors >6 cm, survival was equivalent between radical hysterectomy and radiation. These data suggest that patients with 4–5.9-cm tumors can benefit more from RS than patients with >6-cm tumors.

In this study, the 5-year OS rates were 91.7%, 82.4%, and 57.5% in the lymph node-negative, pelvic lymph node-positive, and para-aortic lymph node-positive groups, respectively. This suggests that patients with lymph node metastases have a worse prognosis than patients with negative lymph nodes, and para-aortic LNM had the worst prognosis. In a prospective study of surgery for stage IB and II CC, Morice et al. reported that the 3-year OS was 94% in patients with negative lymph nodes, 64% in patients with positive pelvic lymph nodes, and 35% in patients with positive para-aortic lymph nodes (P = 0.0001), similar to the results of our study. In addition, we found that patients with positive pelvic or para-aortic lymph nodes had higher overall complications and hematological complications than patients with negative lymph nodes. According to a study by Hua et al., intraoperative and postoperative complications of para-aortic lymph node resection have a serious impact on the quality of life of patients. This suggests that a preoperative evaluation of lymph nodes is important, and surgery should be carefully considered in patients with LNM, especially in the para-aortic lymph nodes.

This study has several limitations. First, this study was a single-arm retrospective study with only an RS group. The survival obtained from single-center studies may be better than that reported in previous studies. Second, some patients were not followed up for 5 years. Compared with the Sedlis criteria, patients with moderate risk, a depth of interstitial invasion in the middle layer, and a negative LVSI did not receive adjuvant therapy before 2015, which may have impacted survival. Finally, follow-up for long-term complications is difficult in retrospective studies, and the rates in the analysis may be lower than the actual rates.

## Conclusion

RS combined with adjuvant therapy is a feasible and effective treatment option for bulky early-stage CC, with a high 5-year survival rate and an acceptable complication rate. Further prospective clinical studies should be carried out to verify the results in the future. Positive pelvic lymph nodes and para-aortic abdominal lymph nodes significantly impact PFS and OS.

## Data availability statement

The raw data supporting the conclusions of this article will be made available by the authors, without undue reservation.

## Author contributions

HT designed, implemented, and revised the article. TZ and PZ designed and directed the experiment. FZ and XT analyzed the data and drafted the manuscript. ZS and ZW conducted the statistical analyses. WG, CF and XC collected clinical and follow-up data. All authors contributed to the article and approved the submitted version.

## Funding

The study was funded by the National Natural Science Foundation of China (81401911) and Medical Science and Technology Project of Zhejiang Province (2021KY555).

## Conflict of interest

The authors declare that the research was conducted in the absence of any commercial or financial relationships that could be construed as a potential conflict of interest.

## Publisher’s note

All claims expressed in this article are solely those of the authors and do not necessarily represent those of their affiliated organizations, or those of the publisher, the editors and the reviewers. Any product that may be evaluated in this article, or claim that may be made by its manufacturer, is not guaranteed or endorsed by the publisher.
